# Diagnostic Value of Lectins in Differentiation of Molar Placentas

**Published:** 2012

**Authors:** Fatemeh Atabaki Pasdar, Alireza Khooei, Alireza Fazel, Mahmoud Mahmoudi, Mohammad Reza Nikravesh, Mohammad Khaje Delui

**Affiliations:** 1 *epartment of Anatomy and Cell Biology, Mashhad University of Medical Science, Mashhad, Iran*; 2 *Department of Pathology, Imam Reza Hospital, Mashhad University of Medical Sciences, Mashhad, Iran*; 3 *Department of Anatomy and Cell Biology, Mashhad University of Medical Sciences, Mashhad, Iran*; 4 *Immunology Research Centre, BuAli Research Institue, Mashhad University of Medical Sciences, Mashhad, Iran*; 5 *Department of Anatomy and Cell Biology, Mashhad University of Medical Sciences, Mashhad, Iran*; 6 *Department of Medical Ethics, Mashhad University of Medical Sciences, Mashhad, Iran*

**Keywords:** Diagnosis, Histochemistry, Hydatidiform mole, Lectins, Spontaneous abortion

## Abstract

**Objective(s):**

Distinction of hydatidiform moles from non-molar specimens and subclassification of hydatidiform moles as complete and partial are important for clinical practice, but diagnosis based solely on histomorphology suffers from poor interobserver reproducibility. Nowadays, pathologists rely on molecular techniques, however these methods are technically difficult, relatively expensive, and time consuming, and cannot be applied in all laboratories. Therefore, a relatively easy, time- and cost-effective ancillary tool, would be helpful. This study aimed to assess the role of lectins in differential diagnosis of molar placentas.

**Materials and Methods:**

Lectin histochemistry with a panel of HRP-conjugated lectins comprising SBA, DBA, MPA, PNA, VVA, UEA-1, LTA, GS-І (B4), and WGA were performed in 20 non-molar (hydropic and non-hydropic spontaneous abortions) and 20 molar (partial and complete moles), formalin-fixed paraffin-embedded tissue samples. On the basis of staining intensity, sections were graded and Kruskal-Wallis non-parametric statistical test was used to compare differences between samples.

**Results:**

There was a significant difference between the reactivities of LTA and UEA-І with syncytiotrophoblasts of molar and non-molar specimens (*P*<0.001). These lectins generally showed a moderate reactivity with syncytiotrophoblasts of molar group but did not react with this cell population in non-molar group. Furthermore, WGA showed relatively increased reaction with syncytiotrophoblasts of molar tissues compared with abortions, however, this did not reach to statistical significance (*P*=0.07). No major differences were seen in other lectins reactivities between the studied groups.

**Conclusion:**

The present study showed that UEA-1 and LTA lectins may be used as cytochemical probes in differentiating molar from non-molar placentas, but did not differentiate partial moles from complete moles.

## Introduction

Gestational trophoblastic disease is a group of interrelated tumors originating from the placenta. Hydatidiform mole is the most common form of, which is an abnormal pregnancy characterized by hydropic swelling of placental villi and trophoblastic hyperplasia; this includes partial hydatidiform mole and complete hydatidiform mole (1).

Placentas characterized by hydropic swelling of chorionic villi occur in a spectrum of pathologic conditions including hydropic abortion, partial hydatidiform mole, and complete hydatidiform mole. Accurate diagnostic classification of hydropic placentas is important as the risk of persistent gestational trophoblastic disease is different among the three entities (2). Whereas hydropic abortion is completely benign, hydatidiform moles have a significant risk for developing persistent gestational trophoblastic disease, with a higher incidence in patients with complete hydatidiform mole (10-30%) than in patients with partial hydatidiform mole (0.5-5%) (3).

Histologic examination is the main tool in the diagnosis of molar pregnancies. However, there is considerable overlap in the histologic features between molar and nonmolar pregnancies and between complete hydatidiform mole (CHM) and partial hydatidiform mole (PHM), resulting in significant interobserver and intraobserver variability in the diagnosis (4, 5). Recently, pathologists have relied on molecular techniques, such as DNA flow cytometry, chromosome *in situ* hybridization, and polymerase chain reaction-based genotyping or HLA typing, which by showing DNA content differences, help to correctly identify the hydropic placentas (6). However, the molecular methods are technically difficult, relatively expensive and time consuming, and cannot be routinely applied in all laboratories. Thus, a time- and cost-effective ancillary tool, available in most laboratories, would be helpful. Lectins are proteins or glycoproteins of nonimmune origin which are extracted from plants and animals. They have been used in many areas of diagnostic investigations, especially those related to changes in the expression of membrane and cytoplasmic glycoconjugates (7, 8). This study aimed to evaluate the use of HRP-conjugated lectins in differential diagnosis of hydropic placentas.

## Materials and Methods

***Case selection***

Formalin-fixed, paraffin-embedded molar and non-molar placental tissue samples of some patients diagnosed in Departments of Pathology of Imam Reza and Qhaem, two teaching hospitals of Mashhad University of Medical Sciences, were collected. Molar tissue specimens consisted of complete hydatidiform mole (n=10) and partial hydatidiform mole (n=10). Non-molar tissue specimens consisted of spontaneous hydropic (n=10) and non-hydropic abortions (n=10). Mean value of gestational age was between 11-12 weeks. Tissue sections of the paraffin blocks were stained with routine hematoxylin-eosin and histopathologically reviewed for confirmation of diagnosis.

***Lectin histochemistry***

The paraffin blocks were cut into serial sections of 4-5 micrometer thickness and some sections were randomly stained with routine hematoxylin-eosin for selection of the best region for lectin histochemistry. Then, the selected sections of each group were deparafinized in xylene and rehydrated through graded dilutions of ethanol. Endogenous peroxidase activity was blocked by preincubation of tissues with 0.5% hydrogen peroxide in methanol for 15 min at room temperature, and then washed in PBS. Tissue sections were covered with HRP-conjugated lectins (SBA, LTA, PNA, MPA, WGA, DBA, GS-І (B4), UEA-1, and VVA), which were purchased from Sigma-Aldrich company and diluted in PBS to reach final concentration of 10 μg of lectins, and placed in a humid chamber for 2 hr at room temperature. The tested lectins and their major sugar specificities are listed in Table 1. After incubation, excess unbound reagent was removed by washing 3 times in PBS and the reaction was then developed in 0.03% in PBS with 0.006% hydrogen peroxidase and after 10 min, reaction was stopped by washing in tap water. The slides were counterstained with alcian-blue (1%) and then dehydrated and mounted in synthetic resin (9-11). 

**Fgure 1:**
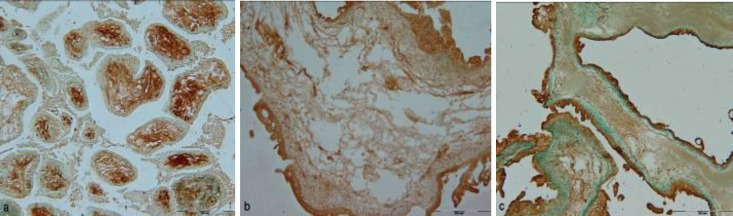
LTA reactivity in non-hydropic spontaneous abortion (a) and complete hydatidiform mole (b), UEA І reactivity in partial hydatidiform mole (c). Scale bar =500 µm

 Negative control samples were made by the same procedure without lectins. For positive controls, human tissues were chosen for each lectin as indicated in the previous publications. Accordingly, normal term placenta was employed as the positive control tissue for WGA (12) and tissue sections of non-neoplastic colonic mucosa for SBA (12), colonic adenocarcinoma for PNA, VVA, and MPA (13, 14) and normal kidney for LTA, DBA, and UEA І (15) were used as positive controls. According to previous studies, in some experiments sialic acid was removed by pretreating the sections for 18 hr at 37 ºC in sodium acetate buffer 0.25 M, pH 5.5, containing 0.1 unit/ml neuraminidase, prior to application of PNA lectin (16, 17). All the slides were stained in the same batch to eliminate inter-batch variation. Ten fields were examined for each section using a light microscope (Olympus AH-2), magnification ×200. The intensity of staining was graded subjectively by two observers. Each observer used a simple intensity scoring system, where negative indicated no staining, weak staining (light brown), moderate staining (brown), and strong staining (dark brown). Kruskal-Wallis non-parametric statistical test was used to compare differences between samples. The level of significance was defined at *P*<0.05.

## Results

Reactivities to the lectins were assessed in villous cytotrophoblasts, syncytiotrophoblasts, and core stromal cells. The results are presented in Tables 2, 3, and 4, respectively.

***Cytotrophoblasts***

DBA reacted with cytotrophoblasts in molar and non-molar tissues, but no significant differences were observed among the studied groups. Other lectins did not react with cytotrophoblasts.

***Syncytiotrophoblasts***

SBA, DBA, VVA, and GS-I (B4) did not react with syncytiotrophoblasts of abortions and molar specimens. PNA also did not react with this cell population, but after neuraminidase treatment (PNA-N), a relatively moderate reactivity was observed in all of groups, which was most pronounced in apical portion. There was a significant difference between the reactivities of LTA and UEA-І with syncytiotrophoblasts of molar and non-molar specimens (*P*<0.001). These lectins generally showed a moderate reactivity with syncytiotrophoblasts of molar group but did not react with this cell population in non-molar group. WGA showed relatively increased reaction with syncytiotrophoblasts of olar tissues compared with abortions, however, this did not reach to a statistical significance (*P*=0.07).

**Figure 2:**
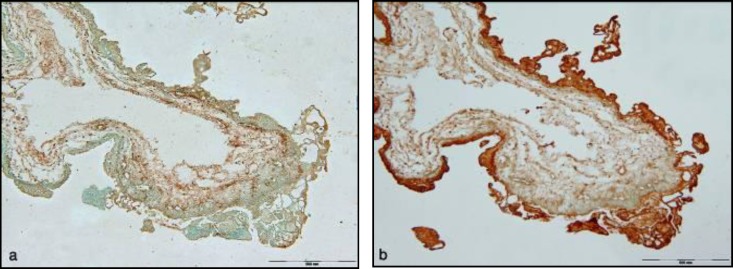
PNA reactivity in complete hydatidiform mole: before neuraminidase digestion (a) and after neuraminidase digestion (b). Scale bar =500µm.

**Table 1:** Lectins used in this study and their major specificities (18, 29, 30)

Name of lectin	Abbreviation	Major sugar specificity
Soybean Agglutinin	SBA	α/β-D-GalNAc > D-Gal
Dolichos Biflorus Agglutinin	DBA	α-D-GalNAc
Maclura Pomifera	MPA	α–Gal , α-GalNAc
Peanut Agglutinin	PNA	D-Gal (β1-3)-D-GalNAc GalNAc
Vicia Villosa	VVA	GalNAc
Ulex Europaeus Agglutinin І	UEA І	α -L-fuc
Lotus Tetragonolobus	LTA	α -L-fuc
Griffonia Simplicifolia	GS-І ( B4)	α–Gal
Wheat Germ Agglutinin	WGA	(GlcNAc)n , Sialic Acid

Abbreviations: GalNAc: N-acetylgalactosamine, Gal: galactose, fuc: fucose, GlcNAc: N-acetylglucosamine

**Table 2:** Intensity of lectins in villous cytotrophoblasts (%)

Lectins	CHM					PHM					HA					Non-HA					*P*-Value
	N	S	M	W		S	M	W	N		S	M	W	N		S	M	W	N		
SBA	100	0	0	0		0	0	0	100		0	0	0	100		0	0	0	100		1.00
DBA	0	10	90	0		10	80	10	0		0	90	10	0		0	100	0	0		0.58
MPA	100	0	0	0		0	0	0	100		0	0	0	100		0	0	0	100		1.00
PNA	100	0	0	0		0	0	0	100		0	0	0	100		0	0	0	100		1.00
PNA-N	100	0	0	0		0	0	0	100		0	0	0	100		0	0	0	100		1.00
VVA	100	0	0	0		0	0	0	100		0	0	0	100		0	0	0	100		1.00
UEA-I	100	0	0	0		0	0	0	100		0	0	0	100		0	0	0	100		1.00
LTA	100	0	0	0		0	0	0	100		0	0	0	100		0	0	0	100		1.00
GS-I(B4)	100	0	0	0		0	0	0	100		0	0	0	100		0	0	0	100		1.00
WGA	100	0	0	0		0	0	0	100		0	0	0	100		0	0	0	100		1.00

Abbreviations: CHM: Complete hydatidiform mole, PHM: Partial hydatidiform mole, HA: Hydropic abortion, Non-HA: Non-hydropic abortion, N: Negative, W: Weak, M: Moderate, S: Strong

**Table 3:** Intensity of lectins in villous syncytotrophoblasts (%)

Lectins	CHM					PHM					HA					Non-HA					*P*-Value
	S	M	W	N		S	M	W	N		S	M	W	N		S	M	W	N		
SBA	0	0	0	100		0	0	0	100		0	0	0	100		0	0	0	100		1.00
DBA	0	0	0	100		0	0	0	100		0	0	0	100		0	0	0	100		1.00
MPA	0	0	80	20		0	0	70	30		0	0	80	20		0	10	70	20		0.85
PNA	0	0	0	100		0	0	0	100		0	0	0	100		0	0	0	100		1.00
PNA-N	300	70	0	0		20	80	0	0		20	80	0	0		20	80	0	0		0.94
VVA	0	0	0	100		0	0	0	100		0	0	0	100		0	0	0	100		1.00
UEA-I	10	90	0	0		0	100	0	0		0	0	0	100		0	0	0	100		<0.001
LTA	20	80	0	0		10	90	0	0		0	0	0	100		0	0	0	100		<0.001
GS-I(B4)	0	0	0	100		0	0	0	100		0	0	0	100		0	0	0	100		1.00
WGA	80	20	0	0		70	20	10	0		30	70	0	0		30	70	0	0		0.07

Abbreviations: CHM: Complete hydatidiform mole, PHM: Partial hydatidiform mole, HA: Hydropic abortion, Non-HA: Non-hydropic abortion, N: Negative, W: Weak, M: Moderate, S: Strong

**Table 4:** Intensity of lectins in villous stromal cells (%)

Lectins	CHM					PHM					HA					Non-HA									*P*-Value	
	S	M	W	N		S	M	W	N		S	M	W	N		S	M		W		N					
SBA	30	70	0	0		30	70	0	0		60	30	10	0		70		20		10		0				0.38
DBA	30	60	10	0		30	70	0	0		60	40	0	0		60		40		0		0				0.26
MPA	0	10	90	0		0	10	80	10		0	0	80	20		0		10		80		10				0.45
PNA	20	80	0	0		10	80	10	0		0	90	10	0		0		90		10		0				0.27
PNA-N	20	80	0	0		10	90	0	0		10	80	10	0		10		90		0		0				0.73
VVA	0	0	0	100		0	0	0	100		0	0	0	100		0		0		0		10				1.00
UEA-I	20	80	0	0		10	90	0	0		0	90	10	0		10		80		10		0				0.35
LTA	0	90	10	0		10	80	10	0		0	90	10	0		10		90		0		0				0.54
GS-I(B4)	0	100	0	0		0	90	10	0		0	90	10	0		0		90		10		0				0.79
WGA	0	20	80	0		0	10	90	0		0	20	80	0		0		20		80		0				0.92

Abbreviations: CHM: Complete hydatidiform mole, PHM: Partial hydatidiform mole, HA: Hydropic abortion, Non-HA: Non-hydropic abortion, N: Negative, W: Weak, M: Moderate, S: Strong

***Core stromal cells***

All of the used lectins except VVA, reacted with core stromal cells. There were no significant differences in the reactivities of lectins between various groups.

## Discussion

The present lectin-binding analysis of hydropic placentas was undertaken to identify possible new diagnostic markers that could be used in differential diagnosis of molar from non-molar hydropic placentas.

There have been several studies on the lectin-binding properties of chorionic villi in normal pregnancies, however, their findings are different (12, 19-21). We found a few studies about lectin-binding properties of molar pregnancies and spontaneous abortions. In the present study, for detection of the fucosyl residue, two different types of lectins, UEA І, and LTA, were employed. Reactivity with LTA suggests the presence of reactive sites containing α-L-fucose which bind via α(1-6) linkage to penultimate glucosaminyl residues and/or difucosylated oligosaccharides (22) while reactivity with UEA І, indicates the presence of α-L-fucose bound via β1,2 linkage to penultimate D-galactose-(β1-4)-N-acetyl-D-glucosamine residues (23). In molar group (partial and complete), LTA and UEA І, reacted moderately with syncytiotrophoblast which was prominent in apical portion, thus revealing the presence of α-L-fucose with both types of linkage, while in non-molar group (hydropic and non-hydropic abortions) no reaction with these lectins was seen in this cell population. The apical portion of the trophoblast corresponds to the microvillous brush border that has been shown to be heavily glycosylated in previous studies (24, 25). The brush border of the syncytiotrophoblast layer of the placenta forms the first barrier separating the maternal blood from the fetal circulation and is important in the exchange of nutrients, hormones, and waste products between the mother and the fetus. It is likely that the syncytiotrophoblast brush border plays a major role in the development of maternal immune tolerance (26). In previous studies on the human normal placenta, neither of these lectins reacted with the trophoblast (19, 20, 27). However, Sgambatti *et al* reported the reaction with LTA and UEA І lectins observed in apical portion of syncytiotrophoblasts of normal placenta which increased during the late stage of placentation, whereas no binding of these lectins was seen in trophoblasts of human placenta of pregnancies complicated by intrauterine growth retardation which suggests the role of α-L-fucose in nutrient transfer (17). In this study, the moderate reactivity of syncytiotrophoblasts with LTA and UEA І, may be due to increased growth and proliferation of trophoblast in molar pregnancies which demand more exchange of nutrients and metabolic products. Our findings are discordant with the study done by Thrower *et al* who detected no binding of UEA І lectins to molar villous syncytiotrophoblast (28). Proteolytic treatment of paraffin sections done in this study affects lectin histochemistry (29), on the other hand, different detection methods may affect the results of lectin histochemistry(they used biotinylated lectins in their studies).

Increased WGA binding in syncytiotrophoblast of molar group compared with non-molar group could be due to increased N-acetylglucosamine and/or sialic acid. However, Tatsuzuki *et al* demonstrated that the brush border of syncytiotrophoblast layer of human term placenta strongly expressed GlcNAc and weakly expressed sialic acid (21). This is consistent with previous study done by Juan *et al* who showed that this increased reactivity was correlated with growth and proliferation of trophoblasts in trophoblastic disease (16). However, their study did not include spontaneous abortions and they used biotinylated lectins. In contrast, Thrower *et al* showed a weak reactivity in syncytiotrophoblast of all examined specimens comprising normal term pregnancy, ectopic pregnancy, and molar pregnancies, using WGA lectins. As described above, this may be due to different detection method or using of proteolytic treatment.

In the present study, as described in some previous studies (21, 30), PNA did not bind with villous syncytiotrophoblast and cytotrophoblast prior to neuraminidase treatment. However, after pretreatment with neuraminidase, the villous syncytiotrophoblasts showed moderate binding with PNA in molar and non-molar groups similar to previous studies (16, 17, 19). PNA lectin has been shown to have specificity for D-Gal (1-3)-D-GalNAc which is supposed to be the antigenic determinant for the Thomsen-Friedenreich antigen or TF-Ag (28, 29). This antigen is normally present in many structures. Ritcher *et al* reported expression of this antigen on trophoblast cells (31), but is considered cryptic, because it is usually covered by a terminal sialic acid. Pretreatment of tissue section with neuraminidase prior to application of PNA lectins will expose this T-Ag as was shown in the normal placenta (16). 

All of the lectins except VVA reacted with villous stroma of both examined groups. This concurs with previous studies done in molar pregnancies (16) and normal placenta (17). However, Lee and Damjanov (19) reported that VVA reacted with stroma of normal placenta.

There were no major differences in MPA, DBA, SBA, VVA, and GS-І (B4) reactivities with cytotrophoblast and syncytiotrophoblast of both molar and non-molar groups.

## Conclusions

The results of this study suggest that lectin histochemistry can be used as an aid in the differential diagnosis of molar from non-molar hydropic placentas. Moreover, UEA-1, LTA and WGA lectins may be used as cytochemical probes in differentiating molar from non-molar placentas, but did not discriminate partial moles from complete moles.
